# A Quantitative Evaluation of the Performance of the Low-Cost AudioMoth Acoustic Recording Unit

**DOI:** 10.3390/s23115254

**Published:** 2023-06-01

**Authors:** Sam Lapp, Nickolus Stahlman, Justin Kitzes

**Affiliations:** Department of Biological Sciences, University of Pittsburgh, 103 Clapp Hall, Fifth and Ruskin Avenues, Pittsburgh, PA 15260, USA; nickstahlman@gmail.com (N.S.); justin.kitzes@pitt.edu (J.K.)

**Keywords:** ARU, automated recording unit, AudioMoth, acoustic monitoring

## Abstract

The AudioMoth is a popular autonomous recording unit (ARU) that is widely used to record vocalizing species in the field. Despite its growing use, there have been few quantitative tests on the performance of this recorder. Such information is needed to design effective field surveys and to appropriately analyze recordings made by this device. Here, we report the results of two tests designed to evaluate the performance characteristics of the AudioMoth recorder. First, we performed indoor and outdoor pink noise playback experiments to evaluate how different device settings, orientations, mounting conditions, and housing options affect frequency response patterns. We found little variation in acoustic performance between devices and relatively little effect of placing recorders in a plastic bag for weather protection. The AudioMoth has a mostly flat on-axis response with a boost above 3 kHz, with a generally omnidirectional response that suffers from attenuation behind the recorder, an effect that is accentuated when it is mounted on a tree. Second, we performed battery life tests under a variety of recording frequencies, gain settings, environmental temperatures, and battery types. We found that standard alkaline batteries last for an average of 189 h at room temperature using a 32 kHz sample rate, and that lithium batteries can last for twice as long at freezing temperatures compared to alkaline batteries. This information will aid researchers in both collecting and analyzing recordings generated by the AudioMoth recorder.

## 1. Introduction

Acoustic monitoring is an increasingly widespread technique for surveying populations of sound-producing species in the field [1,2,3,4]. The growth in acoustic field surveys in recent years has been supported in large part by the development and availability of a variety of inexpensive autonomous recording units (ARUs), devices that are designed to record audio in the field passively without the need for a human surveyor to be present. ARUs generally use battery power and store recordings locally on the device and can be programmed to record ambient sound at predetermined dates and times [5]. Commercial ARUs have been sold for many years by companies including Titley Scientific and Wildlife Acoustics [6,7]. More recently, inexpensive open-source designs based on Raspberry Pi’s [8,9,10] demonstrated that ARUs could potentially be produced more widely and at a far lower cost than previously possible.

In 2017, Open Acoustic Devices released the first version of the AudioMoth [11], which has rapidly become one of the most widely used ARUs, with over 30,000 units produced and sold in the first four years after its release [12]. AudioMoths have been fabricated for as little as USD 50 and have most recently been sold through group purchases for USD 80 [13]. These devices combine many of the user-friendly features of commercial devices at a fraction of the cost.

Despite its popularity, little information is currently available about the performance characteristics of the AudioMoth recorder. From the perspective of audio quality, sensitivity, directionality, and frequency response (relative sensitivity across frequencies) are fundamental characteristics of audio recording equipment that must be measured to interpret audio data captured by a device. Although the frequency response of the AudioMoth’s isolated microphone component is available online [14], the device’s end-to-end on-axis and polar frequency response and sensitivity across gain settings have not been published. This is particularly important as deployment instructions have suggested attaching the AudioMoth to the trunk of a 200–400 mm tree [15], but the effect of this mounting option on audio recordings is not known. These quantitative measurements of recording equipment are distinct from measuring the maximum detection distance of biotic sounds, which depends on a combination of the properties of the recording equipment, the strength and characteristics of the sound source, and environmental noise. Other studies have investigated the maximum detection distance of wildlife using AudioMoth recorders [16,17], and previous literature has described the importance of considering maximum detection distance during the design of acoustic monitoring studies and the analysis of bioacoustics data [18,19].

The recording lifespan of the AudioMoth on one set of batteries has previously been measured only for a subset of the possible configuration settings. Hill et al. reported the battery life of the AudioMoth using 3000 mAh lithium batteries for some common configurations [15], reporting that an AudioMoth could record for 115 days recording at 8 kHz, the lowest sample rate, for 30 s every 5 min. The developers also reported the AudioMoth lasted 9 days recording nonstop at a 48 kHz sample rate. While the AudioMoth configuration app provides estimates of battery life for any chosen configuration settings, these estimates have not to our knowledge been validated empirically. 

Here, we report the results of two sets of tests that we conducted to empirically characterize the performance of the AudioMoth ARU. First, to characterize the acoustic properties of the device and its onboard microphone, we conducted controlled playback tests alongside a test microphone across a variety of device orientations and mounting options. Second, to characterize the longevity of the device in the field, we investigated the expected battery life for the device across a variety of available settings, battery types, and ambient temperatures. This information will assist investigators in designing field experiments using AudioMoth ARUs as well as analyzing the resulting recordings.

## 2. Materials and Methods

Both tests described below were performed using AudioMoth 1.1.0 recorders from 2021 through 2023. Schematics, printed circuit board layout, and components for AudioMoth 1.1.0 are publicly available [15]. Firmware versions varied between the tests and are described below. A full list of all equipment used is compiled in Appendix A. Additional information on these tests and results can be found in [20,21].

### 2.1. Acoustic Performance Test

Our first test had the goal of characterizing the end-to-end sensitivity of the AudioMoth recorder from the microphone through analog-to-digital conversion. To do this, we examined five specific assessments, described below. In all testing and reporting of the results, we follow the guidelines and recommendations of Eargle [22] wherever feasible. All uses of the word “decibels” refer to a logarithmic value and are reported with respect to a reference value. For sound pressure level (a physical measurement of sound in air), dBA is used: the “A-weighted” average of sound pressure level across frequencies, with a reference point of 20 micro-Pascals = 0 dBA [23]. For digital values, dBFS for “decibels full scale” is used, where 0 dBFS is the highest measurement possible in the digital system. In many cases, frequency responses are reported relative to a reference, for instance, relative to the value of the frequency response at 1 kHz, or relative to the on-axis frequency response.

Unless noted otherwise, the gain setting is 0 (low) for all assessments, which allows the greatest difference between the test signal and environmental noise without clipping. The sampling rate was set to 48 kHz and all AudioMoths were using 1.3.0 firmware. The pink noise test signal used throughout the assessments was generated with a Mackie SRM-450 loudspeaker at a level of 86 dBA at one meter in front of the speaker. The AudioMoth was placed 1 m in front of the speaker, except where noted. All frequency responses and analyses use a frequency range of 100 Hz to 17 kHz, as the speaker was unable to reliably reproduce sounds above this range. This range covers the vocalizing range of virtually all birds and frogs and some insects, but not bats [24,25,26,27]. All plots of frequency response show frequency on the *x*-axis on a logarithmic scale from 100 Hz to 17 kHz and decibels (dB) on the *y*-axis with a range of 55 dB so that figures can be compared easily. 

The first assessment compared variations in the frequency response patterns of five new AudioMoths. Comparisons occurred in a 4 × 6 m room in a residential area with acoustically absorbent panels surrounding the microphone to reduce ambient noise levels and reflections. The ambient noise level was measured to be 45.5 ± 0.5 dBA using an Enviro meter EM80 A-weighted Sound Level Meter (Sealed Unit Parts Co., Inc., Allenwood, NJ, USA).

Second, we assessed the effect of the gain setting on frequency response. The AudioMoth has five gain settings, numbered from 0 (low) to 4 (high). For this assessment, pink noise was played at 69 dBA at 1 m to avoid clipping on the highest gain setting of 4. Comparisons occurred in the same room as the first test.

Third, we evaluated on-axis frequency response in a grassland environment using three different AudioMoth protective housing conditions: no case, a Ziploc bag, and a sealed vacuum bag with air. The grassland environment was located at the University of Pittsburgh’s Pymatuning Lab of Ecology Wood Lab site. This environment minimized reflections from buildings and other objects, as the nearest building was over 30 m from the testing location and the speaker faced away from that building. Ambient noise levels recorded before, during, and after testing were 49 ± 3 dBA. 

Fourth, we evaluated the polar pattern, which is the sensitivity of a microphone when the sound arrives from different angles relative to the device. The microphone on the AudioMoth is omnidirectional, meaning that it has equal sensitivity to sound arriving from any direction. However, the device itself is unlikely to be truly omnidirectional, especially for high frequencies, because the device will reflect or absorb some sounds before they reach the microphone. We tested the polar pattern at various frequencies by incrementally rotating the AudioMoth 360 degrees in the horizontal or vertical plane while keeping the source at a fixed position. These tests were performed in the same grassland environment as the on-axis tests. Housing was not found to substantially affect response; therefore, results in different housings are not reported here but can be found in [20].

Fifth, we assessed the acoustic effect of mounting AudioMoth on trees, a common means of deploying devices in forested environments. The forest environment used for testing was a mixed coniferous and deciduous second-growth forest located at the Pymatuning Lab of Ecology housing site. Ambient noise levels recorded before, during, and after testing were 47 ± 1.5 dBA. AudioMoths were strapped to the front, back, and sides of trees from the perspective of the sound source, with the microphone facing away from the tree. We examined three trees with circumferences of 41 cm, 97 cm, and 170 cm. Housing was not found to affect response; therefore, results in different housings are not reported here but can be found in [20].

### 2.2. Battery Life Test

Our second test had the goal of estimating the total number of hours that an AudioMoth can record under a variety of configurations and conditions expected to be encountered in the field. We specifically evaluated the influence of sample rate, device gain, device temperature, battery type, and in some cases their interactions on AudioMoth recording time. All tests were performed in a controlled indoor environment using AudioMoths with 1.5.0 firmware and 64 GB SanDisk Ultra microSDXC cards. For all tests, AudioMoths were programmed to record continuously, and battery life was defined as the number of hours of recordings made successfully before the batteries were unable to continue powering the AudioMoths.

First, we evaluated the effect of the sample rate. A total of twenty-one new AudioMoths were configured to record using 2 (medium) gain at room temperature (21.5 °C) with Procell 2100 mAh alkaline batteries. Three AudioMoths were set to the same sample rate across each available setting (8, 16, 32, 48, 96, 192, 250, and 384 kHz). For comparison purposes, the expected battery life was determined while using the AudioMoth Configuration desktop app by dividing the Procell battery capacity by the daily estimated power consumption. Sample rates above 48 kHz filled the SD card before the batteries died, and cards were replaced as needed to obtain the total estimates of battery life.

Second, we evaluated the effect of gain setting. A total of twelve AudioMoths were configured to record at a 32 kHz sample rate at room temperature with Procell batteries. Three AudioMoths were set to record at the same gain across each available setting (low, low–medium, medium, medium–high, and high).

Third, we evaluated the interacting effects of battery type and temperature on recording time. Procell 2100 mAh alkaline batteries were compared against Energizer Ultimate Lithium 35,000 mAh batteries. Devices with both battery types were placed into three temperature conditions in a consumer-grade refrigerator/freezer combination. We tested battery life using three temperature treatments: room (20.5 °C), fridge (3.4 °C), and freezer (−16.1 °C). The average temperature for each category was determined using an infrared thermometer to obtain readings of the surface next to the AudioMoths during the morning, midday, and evening for each condition and across three separate days. A total of 18 AudioMoths were set to record at a sample rate of 32 kHz with a medium gain. For each battery type (alkaline and lithium), three devices were tested in each temperature condition (room, fridge, and freezer).

## 3. Results

### 3.1. Acoustic Performance Tests

#### 3.1.1. Frequency Response Variation

AudioMoth devices appear to have consistent frequency response patterns with minimal variation. The maximum variation at any frequency between devices was +4.0/−3.5 dBFS, and the overall recording levels were indistinguishable. Small increases or decreases in measured sensitivity at specific frequencies may have been caused by imperfect measurement conditions rather than differences in the devices themselves. Figure S1 shows the frequency response variation of each device relative to one reference device.

#### 3.1.2. Effect of Gain on Frequency Response Variation

The gain settings do not affect the frequency response, besides an overall change in the recorded level. The average levels for gain settings 0, 1, 2, and 3 relative to the highest settings are −14.2, −12.0, −5.7, and −2.6 dB, respectively. Figure S2 plots the frequency response of one device set to each gain setting, relative to the highest gain setting.

#### 3.1.3. On-Axis Frequency Response

**Figure 1:**
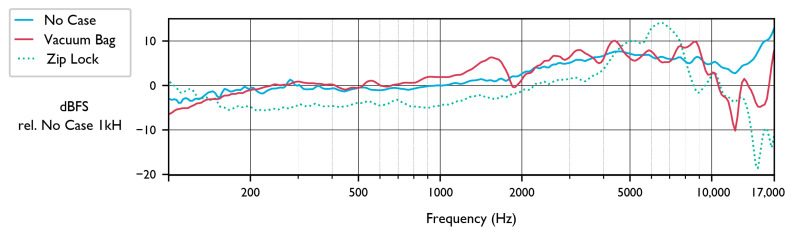
On-axis frequency response of three 1.1.0 AudioMoths recording pink noise playback from 1 m away. One AudioMoth was placed in a closed Ziploc bag, one was placed in a sealed, but not vacuumed, bag, and the control AudioMoth (No Case) was not placed in any housing.

There was wind during on-axis frequency response testing, which registers as low-frequency noise in the recordings. For this reason, the frequency responses reported here may be less accurate below 1 kHz. Figure 1 shows the frequency response of AudioMoth in various protective housings. The 0 dB reference for all lines in this plot is the level of the control, no housing, at 1 kHz. The vacuum bag has the least effect, with the only substantial impact being a 5–10 dB loss above 10 kHz. The frequency response is mostly flat but has sharp dips at around 12 kHz and 15 kHz. The Ziploc bag has slight losses overall, with a bump from 5–10 kHz and a drop-off above 10 kHz. The frequency response of the two housings relative to the control is shown numerically in Table S1.

#### 3.1.4. Polar Response

**Figure 2:**
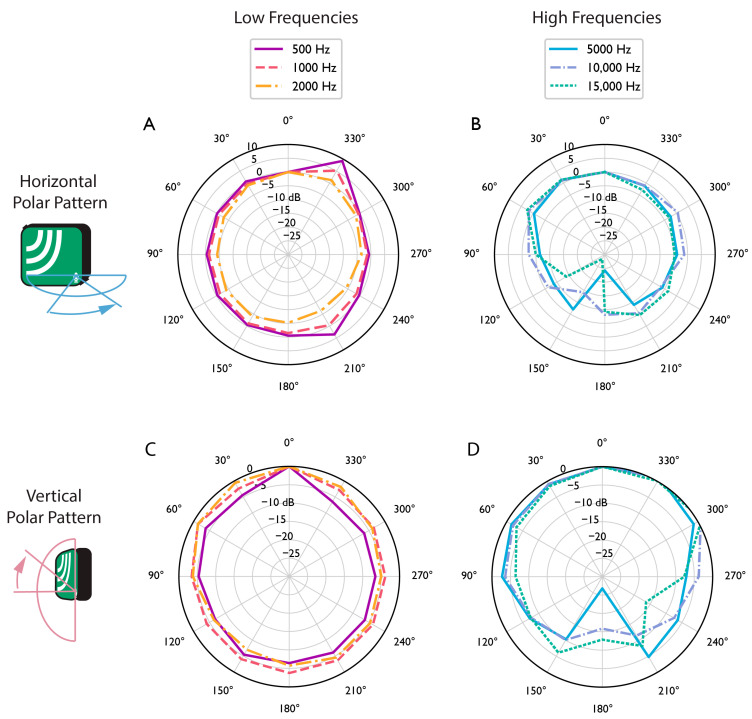
The horizontal polar response of an AudioMoth 1.1.0 with no case is shown at (**A**) 500–2000 Hz and (**B**) 5000–15,000 Hz (90 degrees is to the left and 270 degrees is to the right of the device). The vertical polar response for the same AudioMoth is shown at (**C**) 500–2000 Hz and (**D**) 5000–15,000 Hz (90 degrees is above and 270 degrees is below the device).

As with on-axis testing, there was wind during polar response testing, and the frequency responses reported here may be less accurate below 1 kHz. Figure 2 shows the horizontal polar response of the AudioMoth with no case for several frequencies. Each frequency is plotted in 30-degree increments, relative to that frequency’s level at 0 degrees (on-axis). Lines that dip towards the center of the plot indicate a reduction in sensitivity to that frequency. The polar response is relatively omnidirectional for low frequencies, losing about 5 dB for off-axis sounds. This is expected because sound waves below 2 kHz have wavelengths significantly longer than the dimensions of the device (a 2 kHz sound wave is approximately 17 cm) and are minimally attenuated or reflected by the device. Higher frequencies show significant attenuation directly behind the device, especially at 5 kHz (over 25 dB reduction). The largest effects are losses of 28 dB at 5 kHz and 20 dB at 10 kHz when sound arrives from behind the device. Figure 2 also shows the vertical polar response where the sound was coming from “above” and “below” the AudioMoth. The vertical rotation of the AudioMoth has little effect on low frequencies other than a surprising boost of 500 Hz arriving from slightly below the device. This could be due to a resonance mode in the device or may be a measurement error. In the high frequencies, 15 kHz is severely reduced when it arrives from behind and slightly above the device. This may be a result of cancellation or absorption by the battery pack (15 kHz has a wavelength of about 2 cm).

#### 3.1.5. Impact of Trees on Frequency Response

**Figure 3:**
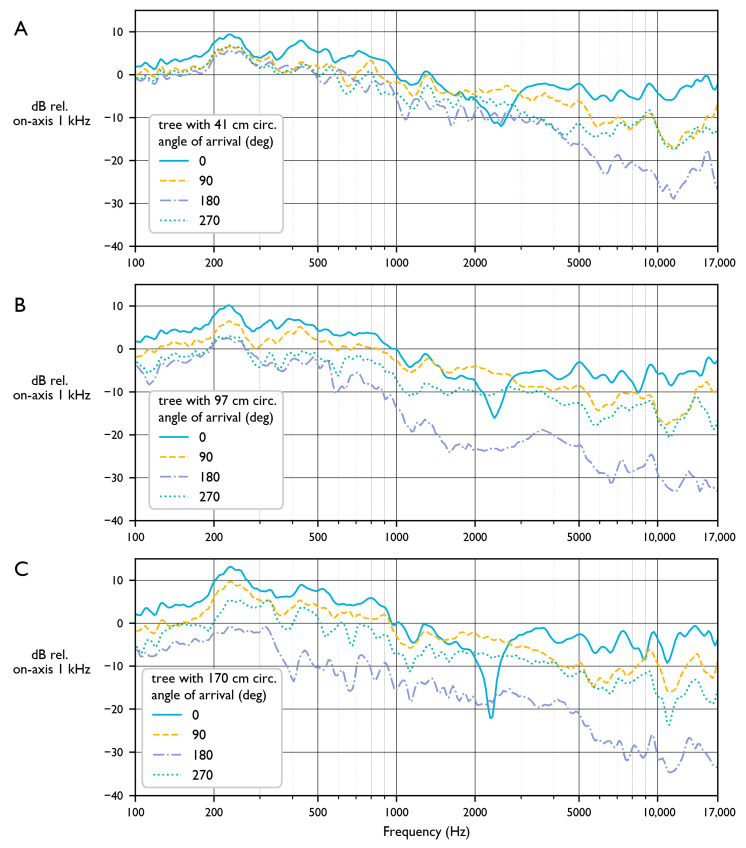
Pink noise recorded by three 1.1.0 AudioMoths that were strapped 1 m away from the playback speaker to the front (0°), back (180°), and sides (90° and 270°) of trees with various circumferences: (**A**) 41 cm, (**B**) 97 cm, and (**C**) 170 cm.

Figure 3 shows the frequency spectrums of pink noise playback tests recorded by an AudioMoth with no case strapped to the front, back, or side of each tree from the perspective of the sound source. When the AudioMoth is on the same side as the tree as the sound source (0 degrees), a narrow notch filter (reduction in sensitivity) appears at 2.3 kHz, indicating cancellation of 15 cm sound waves. One possible cause of this is a reflection of incoming sound off of the cambium of the tree: if sound travels 3.75 cm to the reflective surface and 3.75 cm back to the receiver, it will travel half a wavelength (7.5 cm) round trip and cause destructive interference of 2.3 kHz sound at the microphone. Interestingly, if this is true, this “dead spot” will only have a strong effect directly in front of the microphone and at a specific frequency, as varying the angle of incidence will change the center frequency of the notch filter. Besides the dramatic notch filters, the effects of the trees on frequency response match our expectations. When sound arrives from behind the tree, attenuation increases with frequency and with the size of the tree.

For the smallest tree (41 cm circumference), high frequencies are substantially reduced while low frequencies are unaffected. With increasing tree radius, overall attenuation increases while the pattern of more attenuation at higher frequencies remains.

### 3.2. Battery Life Tests

#### 3.2.1. Effect of Sample Rate

The average hours of audio recorded by each AudioMoth at varying sample rates is summarized in Table 1. There was little variation in recording performance between devices, and replicate performance is shown in Table S2. Under what could be considered baseline conditions, an AudioMoth records an average of 189 h of audio at 20.5 °C with Procell batteries at a 32 kHz sample rate, very close to the 187 h estimated by the AudioMoth configuration app. As expected, there is a negative relationship between the sample rate and the hours of audio the AudioMoth will record. In general, we found that the AudioMoth Configuration App underestimates recording time at low frequencies and overestimates recording time at high frequencies.

#### 3.2.2. Effect of Gain

The gain setting had a negligible effect on the total hours of audio recorded. The average across settings varied by a maximum of 2%. Individual performance and the average is shown in Table S3.

#### 3.2.3. Effect of Battery Type and Temperature

The Procell and lithium temperature trials are summarized in Table 2. For Procell batteries, there is a small 3% decrease in hours recorded between the control devices at room temperature (20.5 °C) and the devices at 3.4 °C. In contrast, devices at −16.1 °C recorded just more than half as long as those at room temperature. The lithium batteries had relatively consistent recording times across all temperatures, all of which were higher than the recording times for the Procell batteries. With lithium batteries, the AudioMoths recorded for a slightly longer time at colder temperatures compared to room temperature. Results were relatively consistent across devices as shown in Tables S4 and S5.

## 4. Discussion

### 4.1. Acoustic Performance Tests

In summary, our results show that the AudioMoth has favorable acoustic recording properties and battery life. The frequency response was found to be generally flat, with a slight increase in sensitivity above 3 kHz, peaking between 5 kHz and 10 kHz (Figure 1). The AudioMoth’s frequency response does not vary significantly between devices (Figure S2). Changing the gain settings results in an overall change in recorded signal level, providing a total range of adjustment of 14.2 dB, and changing the gain setting does not affect the relative sensitivity across frequencies. Plastic bags used as weatherproof housing were found to attenuate frequencies above 10 kHz (Figure 1). This result is congruent with physical acoustics, which predicts that sound transmission loss through a membrane will be low for thin, flexible membranes, but will increase with frequency [28]. The polar response pattern of the AudioMoth showed that the device has lower sensitivity behind the device than in front, an effect accentuated when the recorder was mounted on a tree. This lack of omnidirectionality should be considered during data analysis, especially when estimating the area surveyed by a recorder or estimating the distance to a sound source. During deployment, attenuation of sounds arriving from behind the device can be minimized by mounting the AudioMoths on trees or stakes with small diameters (3–7 cm). We note that all tests were completed using new devices that had not previously been deployed in the field, and that acoustic performance may be affected by microphone damage after devices are left in the field for extended periods [29].

In practice, the AudioMoth is rarely used without protective housing and is often attached to a tree. When planning deployments of AudioMoths for field data collection, the effects of such choices should be considered. If a deployment strategy will cause substantial reductions in certain frequency bands, the downstream effects of this lost information should be carefully considered. For instance, strapping an AudioMoth to a 30 cm diameter tree will result in a sensitivity to high frequencies that is about 25 decibels higher in front of the device than behind the device. Considering that sound decays approximately 6 dB per doubling of distance ignoring absorption and attenuation, the maximum recording distance of an event behind the device would effectively be four times smaller than the front. 

Our results show that even when the AudioMoth is not in a case and not strapped to a tree, it is not truly omnidirectional. When sound arrives from behind the microphone, there is an overall reduction of at least 5 dB for all frequencies, and certain frequencies (around 5 kHz and 10 kHz) are sharply reduced. A reduction of level in a frequency response plot can be thought of as a loss of information for each frequency. While an overall loss of level simply results in quieter files (and effectively a smaller sampling radius), the loss of specific frequencies more than others “distorts” the data by recording some sounds quieter than others of equivalent volume. This would be problematic if, for instance, a study was to attempt to compare the presence of two species with vocalizations in two specific frequency ranges, one of which was recorded with 15 dB less sensitivity than the other. During analysis, it is important to account for any frequency-dependent sensitivity of the deployment strategy.

Cases made of solid materials will have different effects on frequency response than the thin, flexible bags used for housing in this report. Sound travels easily through flexible membranes but is absorbed or reflected by thick solid membranes [28]. Higher frequencies will be attenuated more than lower frequencies when traveling across a solid membrane, and overall attenuation will be correlated with the thickness of the membrane [28]. Because the precise effects of a protective housing on the recorded audio are difficult to predict, the effect of alternative housings on frequency response should be measured before deployment.

When choosing a gain setting for an AudioMoth deployment, the goal is to choose the highest gain setting that does not cause clipping. Clipping occurs when the signal level exceeds the maximum levels that can be recorded, and causes harmonic distortion of the audio signal, which results in the introduction of harmonics not present in the real-world signal. Because field sites and organisms can differ greatly in noise levels, experimenting with different gain settings at a specific field site is the best way to determine an appropriate gain setting that maximizes recording levels while avoiding clipping. 

### 4.2. Battery Life Tests

In summary, our results show that recording times decreased with an increasing sample rate, as expected. Expected recording times predicted by the AudioMoth configuration app for alkaline batteries were consistent with our results for sampling rates of 32 kHz. The configuration app underestimated recording times at lower sampling rates and overestimated recording times at higher sampling rates. When planning field deployments around AudioMoth battery life, we recommend using the results of our empirical experiments rather than the configuration app’s estimates. As expected, cold temperatures decreased recording times with alkaline batteries, although this effect was not substantial until all temperatures fell below 0 °C. Lithium batteries were recorded for similar amounts of time at all three examined temperatures.

## 5. Conclusions

Our tests show that the AudioMoth’s long battery life, directional pattern, and flat frequency response make it an effective recording hardware choice for bioacoustic monitoring. The recorder has a relatively flat frequency response and records sound from all directions effectively, although we note that the device is not fully omnidirectional. We found that plastic bags have little effect on sensitivity to frequencies below 10 kHz, making them ideal housings in environments where they provide sufficient protection. When using AudioMoths or any other automated recording unit, the characteristics of the recording hardware and housing should be considered during data analysis. In particular, the frequency response and directionality of the recorder, and the effect of mounting the recorder on solid objects such as trees, should be considered when evaluating recordings. The frequency response measurements and battery life tests provided in this report can be used to inform the survey design and data analysis of acoustic recordings collected by AudioMoths.

## Figures and Tables

**Table 1:** The average, minimum, and maximum hours of audio recorded across three 1.1.0 AudioMoths at each sample rate and the average amount of data written to the SD cards compared against the AudioMoth configuration app estimations.

Sample Rate (kHz)	Hours Recorded			Configuration App Estimate (h)
	Mean	Min	Max	
8	249	247	251	229
16	224	224	225	210
32	189	187	191	187
48	161	160	163	168
96 ^1^	91	90	91	133
192 ^1^	60	59	61	87
250 ^1^	47	47	47	80
384 ^1^	43	43	43	55

^1^ Audio files at these sample rates filled the SD card before the batteries depleted, and required a second SD card to be inserted once the red and green LEDs began flashing.

**Table 2:** A comparison of the average, minimum, and maximum hours of audio recorded by 1.1.0 AudioMoths set to record with a 32 kHz sample rate at different temperature ranges using three Procell alkaline or Energizer lithium AA batteries.

Temperature	Procell			Lithium		
	Avg	Min	Max	Avg	Min	Max
Room (20.5 °C)	189	187	191	234	228	239
Fridge (3.4 °C)	183	181	185	241	239	244
Freezer (−16.1 °C)	103	99	105	238	236	241

## Data Availability

All data and results are available in GitHub repositories at https://github.com/kitzeslab/audiomoth-performance (accessed on 27 May 2023) and https://github.com/kitzeslab/ARU_battery_longevity (accessed on 27 May 2023).

## Supplementary Materials

The following supporting information can be downloaded at: https://www.mdpi.com/article/10.3390/s23115254/s1, Figure S1: Frequency response variation between five AudioMoth devices without cases relative to Device 1017; Figure S2: Frequency response for pink noise recorded at the 5 gain settings, relative to the highest gain setting; Table S1: On-axis frequency response in each housing treatment; Table S2: Individual results and overall average for the effect of sample rate on battery life.; Table S3: Individual results and overall average for the effect of gain setting on battery life.; Table S4: Individual results and overall average for the effect of temperature on Procell alkaline battery life; Table S5: Individual results and overall average for the effect of temperature on Energizer lithium battery life.

## Appendix A

Acoustic Performance Test Equipment List

Enviro meters A-weighted EM80 Sound Level (Sealed Unit Parts Co., Inc., Allenwood, NJ, USA)

- AudioMoth version 1.1.0 with 1.3.0 firmware (LABmaker, Berlin, Germany)
- 64 GB SanDisk Ultra microSDXC UHS-I Card (SanDisk, Milpitas, CA, USA)
- dbx RTA-M Reference Microphone (HARMAN International, Stamford, CT, USA)
- Scarlett 2i2 Audio Interface (Focusrite, High Wycombe, United Kingdom)
- Macbook Pro 2013 with Logic Pro X and Audacity 2.2.2 (Apple, Cupertino, CA, USA)
- Mackie SRM-450 Loudspeaker (Mackie, Woodinville, WA, USA)

Battery Life Test Equipment List

- AudioMoth version 1.1.0 with 1.5.0 firmware (LABmaker, Berlin, Germany)
- 64 GB SanDisk Ultra microSDXC UHS-I Card (SanDisk, Milpitas, CA, USA)
- Procell PC1500 Alkaline AA Batteries (Duracell Inc., Chicago, IL, USA)
- Energizer Ultimate Lithium AA Batteries (Energizer Holdings, Inc., St. Louis, MO, USA)
- Frigidaire Top Freezer Refrigerator Model: FFET1022UV (Frigidaire, Charlotte, NC, USA)
