# Supplementary material for: A Quantitative Evaluation of the Performance of the Low-Cost AudioMoth Acoustic Recording Unit
Source: Sensors (Basel). 2023 Jun 1;23(11):5254. doi: 10.3390/s23115254 (PMC10256106; doi:10.3390/s23115254)
Supplement: Supplementary file 1 [file sensors-23-05254-s001.zip › sensors-2410301-supplementary.pdf]

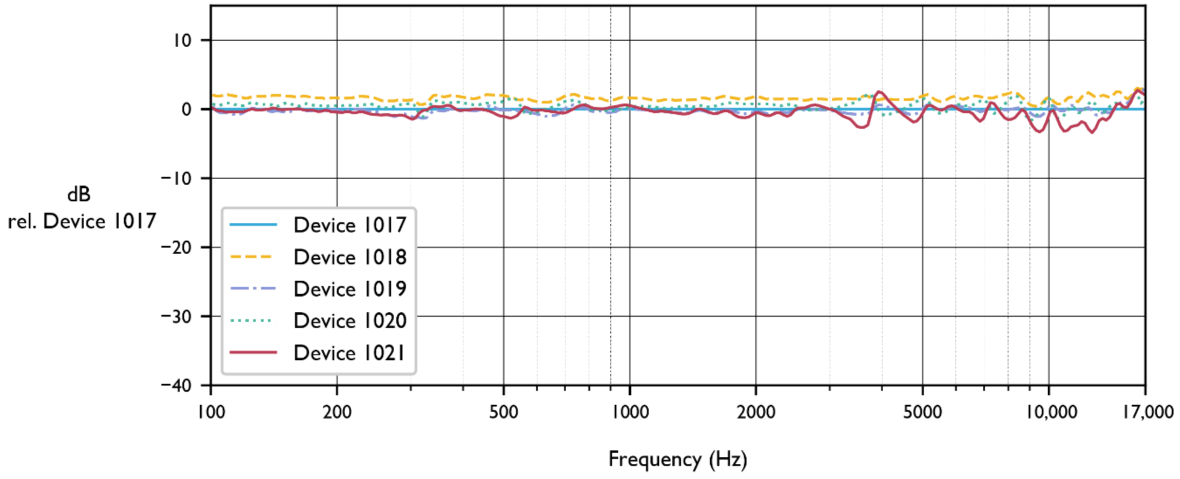


**Figure S1.** Frequency response variation between five AudioMoth devices without cases relative to Device 1017.

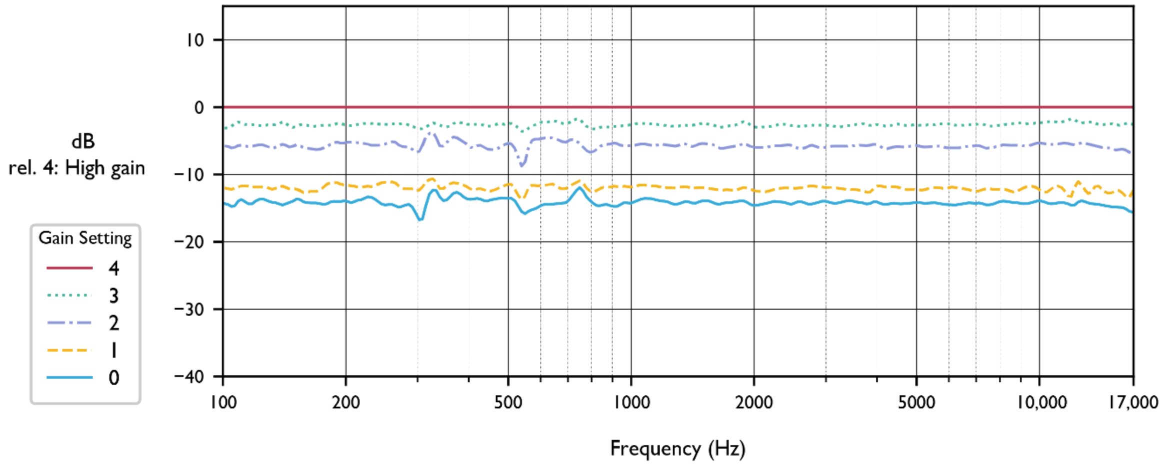


**Figure S2.** Frequency response for pink noise recorded at the 5 gain settings, relative to the highest gain setting.

**Table S1.** On-axis frequency response in each housing treatment, dB relative to "A: No Case" at 1kHz = 0dB

| Treatment | 100 Hz | 200 Hz | 500 Hz | 1 kHz | 2 kHz | 5 kHz | 10 kHz | 15kHz |
|---|---|---|---|---|---|---|---|---|
| A: No Case | −3 | 0 | 0 | 0 | 2 | 7 | 5 | 8 |
| B: Vacuum Bag | −6 | −1 | 0 | 1 | 1 | 5 | 2 | −4 |
| C: Zip Lock | 0 | −4 | −3 | −4 | −1 | 9 | 1 | −15 |

**Table S2.** Individual results and overall average for the effect of sample rate on battery life.

| Sample Rate (kHz) | AudioMoth 1 Hours of Audio | AudioMoth 2 Hours of Audio | AudioMoth 3 Hours of Audio | Average Hours of Audio |
|---|---|---|---|---|
| 8 | 247 | 249 | 251 | 249 |
| 16 | 224 | 225 | 225 | 224 |
| 32 | 187 | 191 | 188 | 189 |
| 48 | 163 | 161 | 160 | 161 |
| 96[1] | 90 | 91 | 91 | 91 |

| | | | | |
|---|---|---|---|---|
| 192[1] | 61 | 59 | 61 | 60 |
| 250[1] | 47 | 47 | 47 | 47 |
| 384[1] | 43 | 43 | 43 | 43 |

[1] Audio files at these sample rates filled the SD card before the batteries depleted, and required a second SD card to be inserted once the red and green LEDs began flashing.

**Table S3.** Individual results and overall average for the effect of gain setting on battery life.

| Gain Setting | AudioMoth 1 Hours of Audio | AudioMoth 2 Hours of Audio | AudioMoth 3 Hours of Audio | Average Hours of Audio |
|---|---|---|---|---|
| Low | 186 | 190 | 187 | 188 |
| Low-medium | 187 | 188 | 190 | 188 |
| Medium | 187 | 191 | 188 | 189 |
| Medium-high | 187 | 186 | 186 | 186 |
| High | 187 | 191 | 187 | 188 |

**Table S4.** Individual results and overall average for the effect of temperature on Procell alkaline battery life.

| Temperature | AudioMoth 1 Hours of Audio | AudioMoth 2 Hours of Audio | AudioMoth 3 Hours of Audio | Average Hours of Audio |
|---|---|---|---|---|
| Room (20.5°C) | 187 | 191 | 188 | 189 |
| Fridge (3.4°C) | 184 | 181 | 185 | 183 |
| Freezer (−16.1°C) | 105 | 103 | 99 | 102 |

**Table S5.** Individual results and overall average for the effect of temperature on Energizer lithium battery life.

| Temperature | AudioMoth 1 Hours of Audio | AudioMoth 2 Hours of Audio | AudioMoth 3 Hours of Audio | Average Hours of Audio |
|---|---|---|---|---|
| Room (20.5°C) | 228 | 236 | 239 | 234 |
| Fridge (3.4°C) | 244 | 239 | 241 | 241 |
| Freezer (−16.1°C) | 241 | 236 | 237 | 238 |
